# The relationship between low back pain and professional driving in young military recruits

**DOI:** 10.1186/s12891-018-2037-3

**Published:** 2018-04-10

**Authors:** Oren Zack, Regina Levin, Ayala Krakov, Aharon S. Finestone, Shlomo Moshe

**Affiliations:** 1 The Israel Defense Forces, Medical Corps, Ramat Gan, Israel; 2 Sackler Faculty of Medicine, School of Public Health, Department of Environmental and Occupational Medicine, Tel Aviv University, Tel Aviv, Israel; 3 Maccabi Healthcare Services, the Occupational Department, Holon, Israel; 4 Department of Orthopedics, Assaf Harofeh Medical Center, Zerifin, Affiliated to the Sackler Faculty of Medicine, Tel Aviv University, Tel-Aviv, Israel; 5 Department of Occupational, 43 Geulim St, Holon, Israel

**Keywords:** Low back pain, Incidence, Prevalence, Occupational exposure, Risks, Driving, Driver, Young adults, Army recruits

## Abstract

**Background:**

Episodes of low back pain (LBP) are very common among workers. A number of occupational risk factors have been shown to increase the risk for LBP. One of these risk factors is exposure to whole body vibration, which is a known characteristic in driving professions. The aim of this study was to assess the impact of driving on LBP amongst young professional drivers.

**Methods:**

This is an historical-prospective cohort study based on the Israel Defense Forces (IDF) database of male soldiers drafted between the years 1997–2006. Subjects’ medical history with specific reference to LBP medical history, clinical and radiographic findings were taken as part of the recruitment process to the IDF. The study group included subjects (*n* = 80,599) from three occupational groups: administrative units (AU), car drivers (CD) and truck drivers (TD) that were followed for 3 years. The incidence and recrudescence rates of LBP were calculated based on standardized LBP severity tiers.

**Results:**

The total incidence rate for LBP was 0.65%, 0.7% and 0.34% for AU, CD and TD respectively. In a comparison between subjects without a history of LBP (category 1) to subjects with a history of LBP without clinical findings (category 2) and subjects with a history of LBP with mild clinical / radiographic findings (category 3), the relative risk (RR) for severe LBP exacerbation was 1.4 (*p* < 0.001) and 3.8 (*p* < 0.01), respectively. The LBP exacerbation rates within different severity tiers yielded a similar trend amongst all profession groups.

**Conclusions:**

This study included 80,000 soldiers who represent a population of driving and administrative occupations aged 18–21. The significant risk factors for developing LBP were a previous history of LBP and presence of LBP symptoms at the start of work. A correlation was identified between severity of LBP at the initial examination and further exacerbation of LBP in all examined occupations. Driving as a profession in our (young) age-group was not identified as a risk factor for LBP.

## Background

Episodes of low back pain (LBP) are very common among workers [1]. LBP causes more global disability than any other condition and ranks highest in terms of disability (Years Lived with Disability -YLD) and sixth in terms of overall burden (Disability Adjusted Life Years - DALY) [2]. In the systematic analysis for the Global Burden of Disease 2010 Study, the global point prevalence of LBP was 9.4% (95% CI 9.0 to 9.8) and in some areas of the world as high as 15.0% (Western Europe – 15.0%, North Africa/Middle East – 14.8%) [3]. The main biomechanical risk factors identified for the development of LBP at work are heavy physical work, awkward static and dynamic working postures, whole body vibration, and lifting [4, 5]. High prevalence of back pain, early degenerative changes of the spine and herniated lumbar disc problems and sciatica, have been consistently reported among vibration-exposed occupational groups [5–7]. The suggested pathological mechanism of vertebral damage due to vibration is induction of micro-fractures at the end-plates, with callus formation during healing. The risk of developing back injuries among truck drivers (TD) is reported in the range of RR = 1.7–3.7 [4, 8]. Several studies, conducted among different driving professions like taxi drivers [9–11], bus drivers [11–13] and TD [14–16], pointed out the relationship between LBP and driving, especially occupational exposure to vibration or road shock.

Military service in the Israel Defense Forces (IDF) is mandatory at the age 18 except for some subpopulations who are exempt from service. It has already been shown that many individuals enter military service with undiagnosed LBP or mild LBP, which consequently limits their functional skills during service [17]. Military service could be challenging for individuals with LBP due to exacerbating factors common in the military environment amongst which is professional driving [17–19].

In our previous work on IDF army soldiers we have shown an overall LBP incidence rate of 0.05% with a higher relative risk of developing LBP amongst administrative occupations which included amongst are driving professions [20]. There is relatively little information about the impact of driving on LBP at young age and its relationship to a known history of LBP. This study aims to provide more insight into the effect of driving on LBP amongst professional drivers early in their military career and the effect of LBP history as an exacerbating risk factor.

## Methods

### Data source

This is an historical-prospective cohort study, largely based on the same data source outlined in our previous study [20], of 18-year-old male soldiers drafted to the IDF between January 1st 1997 through December 31st 2006, that were followed up for 36 months. Subjects’ medical history, including specific reference to LBP medical history, clinical and radiographic findings including medication use, X-ray, CT scans, MRI, EMG etc. were taken by a certified physician as part of the medical profiling used during the recruitment process to the IDF. Referral to a certified orthopedic surgeon, when germane, was part of the examination process during recruitment to the IDF. Each recruit’s medical and examination records were reviewed by a board of military physicians to establish and approve a functional medical-category classification. Inclusion criteria included all subjects that served in administrative or driving professions for a full 36-months period during the study period of Jan 1st 1997 – Dec 31st 2006.

### Population occupational groups

The current study focuses on soldiers from three occupational groups: administrative units (AU) engaged in mostly sedentary work (considered as the control group), professional car drivers (CD) and professional truck drivers (TD). Both groups of drivers (CD and TD) undergo similar basic training and are engaged in daily driving tasks which differ mainly in extent and type of vehicle: car drivers typically drive short trips within municipal areas using passenger vehicles whereas truck drivers drive longer, inter-city, trips in heavy vehicles (trailers, and semi-trailers).

Assignment of soldiers into the above groups was based on the varying demands and tasks for each occupation and the matched functional medical-category classification. As a rule, administrative professions require the lowest medical threshold and include subjects from all medical tiers whereas combat professions require the highest medical threshold and include low-tiers only. Driving professions are middle-tiered with TD exhibiting a higher threshold than CD.

### Population medical categories

Medical categories used in our current study are based on a tier system from the IDF’s book of medical profiles which are based on reported LBP history, findings on physical examination and radiologic findings (X-ray/CT/MRI) [20]. Four code-based categories were included in our study: Category 1 – No history of LBP, Category 2 – Mild LBP, Category 3 – LBP with mild clinical/radiographic findings and Category 4 (representing the study endpoint and a dependent variable) – LBP with substantial clinical and correlating radiographic findings. Category 4 soldiers are determined unfit for driving professions and are hence reassigned accordingly. By definition of the inclusion criteria above and the minimum fitness criteria for driving professions, it should be noted that soldiers already meeting category 4 severity at recruitment, were not included in the study.

### Follow up during the service (Fig. 1)

All subjects were followed for 36 months by the units’ physicians noting symptoms of LBP. In cases of newly reported or exacerbating symptoms, the subjects were reevaluated by a certified orthopedic surgeon in accordance with the medical parameters defined in the military medical book of profiles. The medical status was then reassessed by a military medical committee comprised of two senior physicians. When applicable, the medical category was adjusted according to the new evidence. Exacerbation, representing the study endpoint per subject, was defined as a change in the medical category from either preliminary categories 1–3 to category 4. Due to lack of relevance to the study objectives, exacerbations from category 4 to more severe categories are not presented.

**Fig. 1:**
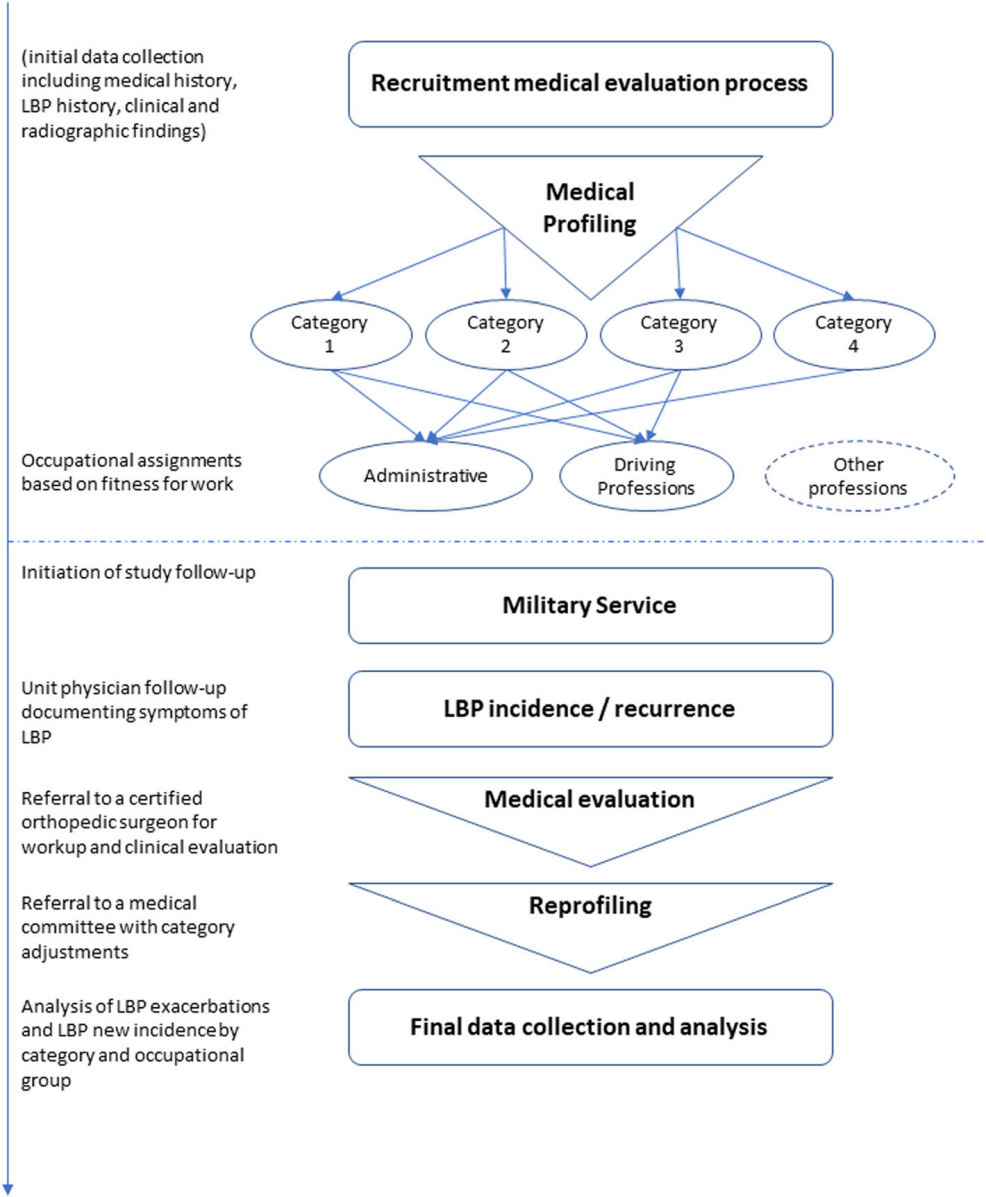
Depicts a flowchart of the medical evaluation process and the study endpoints starting from the point of recruitment to occupational assignments and follow-up period in an effort to document new incidence and exacerbations of LBP

### Data analysis

Incidence and relative risk rates were compared using stratified analysis to assess up-tiering (higher severity), according to disease categories. A two-tailed *p* < 0.05 was calculated using Fisher’s exact test, and considered significant when calculating relative risks (RR) and confidence intervals for new onset LBP during the study period. All analyses were conducted using a standard statistical package (Compare2 version 2.97, Copyright JH Abramson 2000–2001).

### Ethics approval and consent to participate

Ethical approval was obtained from the IDF medical corps ethics committee (reference number 1175–2012). The IDF ethics committee waived the need for formal informed consent since actual patient files were not reviewed in this study but rather a computerized database of coded information (whereas medical profiling involves a physician-patient encounter, our study was based only on outcomes (tier classification) obtained from such encounters).

## Results

The study population included 80,599 soldiers who met the inclusion criteria. The average age of the cohort was 19.06 ± 1.4 years. Table 1 summarizes the annual incidence rates of LBP during the study period according to the subject’s status at recruitment. Higher-severity categories exhibit higher incidence (exacerbation) rates. The crude incidence rates for LBP among categories 1 to 3 were found to be 0.65%, 0.90% and 2.49%, respectively. Amongst category 1 subjects, the incidence of newly diagnosed LBP was significantly lower amongst TD compared to either AU or CD (RR = 0.49, *p* < 0.0001).

**Table 1:** Relative risk (RR) and 95% confidence intervals (CI) for newly diagnosed and exacerbated low back pain (LBP) among soldiers (18–21 years), by severity of disease and occupational category

Severity of LBP at recruitment	Occupational Categories	Incident cases (IR*) for 3 years	Incident cases (IR*) per year	RR (95% CI)	*P*
Category 1 (No history of LBP)	Administrative (*n* = 61,046)	2.09%	0.7%	1	
	Car drivers (*n* = 3254)	2.09%	0.7%	1.0 (0.79–1.28)	0.957
	Truck drivers (*n* = 9689)	1.02%	0.34%	0.49 (0.40–0.60)	**< 0.0001**
Total	*n* = 73,989	1.95%	0.65%	–	–
Category 2 (Mild LBP)	Administrative (*n* = 4612)	2.67%	0.89%	1	
	Car drivers (*n* = 333)	3.00%	1.00%	1.13 (0.60–2.12)	0.724
	Truck drivers (*n* = 743)	2.75%	0.92%	1.03 (0.5–1.81)	0.881
Total	*n* = 5418	2.69%	0.90%	–	–
Category 3 (LBP with mild clinical/radiographic findings)	Administrative (*n* = 1066)	7.41%	2.47%	1	
	Car drivers (*n* = 60)	10.00%	3.33%	1.35 (0.61–2.97)	0.448
	Truck drivers (*n* = 66)	6.06%	2.02%	0.82 (0.31–2.16)	1.000
Total	*n* = 1192	7.47%	2.49%	–	–

Bold data already signifies a *p* < 0.05 i.e. significant finding

Intra-category comparison of incidence rates by profession yielded no significant findings for either category 2 or 3.

Table 2 exhibits exacerbation rates within the varying occupational groups according to initial severity of LBP (categories 1–3). Examination of RR exacerbation rates within different severity tiers yielded a similar trend amongst all occupational groups (AU, CD and TD) with an increased risk when comparing higher categories (3 and 2) to initial category at recruitment.

**Table 2:** Relative risk (RR) and 95% confidence intervals (CI) for exacerbation of low back pain (LBP) within the occupational groups by severity of disease at recruitment

Severity of LBP at recruitment by occupational group	Number exacerbated (total number)	Incident cases (IR) for 3 years	Incident cases (IR) per year	RR (95% CI)	*P*
Administrative					
Category 1	1274 (61046)	2.1%	0.7%	1	
Category 2	123 (4612)	2.7%	0.9%	1.28 (1.07–1.53)	**< 0.01**
Category 3	79 (1066)	7.4%	2.5%	3.55 (2.85–4.42)	**< 0.0001**
Car drivers					
Category 1	68 (3254)	2.1%	0.7%	1	
Category 2	10 (333)	3.0%	1.0%	1.48 (0.75–2.76)	0.32
Category 3	6 (60)	10.0%	3.3%	4.79 (2.16–10.59)	**< 0.002**
Truck drivers					
Category 1	99 (9689)	1.0%	0.3%	1	
Category 2	13 (473)	2.7%	0.9%	2.69 (1.52–4.76)	**< 0.002**
Category 3	4 (66)	6.1%	2.0%	5.93 (2.25–15.64)	**< 0.005**
Total drivers					
Category 1	167 (12943)	1.3%	0.4%	1	
Category 2	23 (806)	2.9%	1.0%	2.21 (1.44–3.40)	**< 0.001**
Category 3	10 (126)	7.9%	2.6%	6.15 (3.33–11.36)	**< 0.0001**
All subjects					
Category 1	1441 (73989)	1.95%	0.65%	1	
Category 2	146 (5418)	2.69%	0.90%	1.38 (1.17–1.64)	**< 0.0001**
Category 3	89 (1192)	7.47%	2.49%	3.83 (3.12–4.71)	**< 0.0001**

Category 1 – No history of LBP

Category 2 – Mild LBP

Category 3 – LBP with mild clinical/radiographic findings

Bold data already signifies a *p* < 0.05 i.e. significant finding

It can also be seen that for the combined total drivers group, the same linear and significant relationship was found between initial category at recruitment and the risk for LBP exacerbation during the study period with incidence rates of 0.4%, 1.0% (RR = 2.21, *p* < 0.001) and 2.6% (RR = 6.15, *p* < 0.0001) for categories 1–3, respectively.

The calculated RR for exacerbation to severe LBP for soldiers with a history of LBP without clinical findings was RR = 1.38 (p < 0.0001) and even higher for those with a history of LBP with mild clinical/radiographic findings RR = 3.83 (p < 0.0001).

Table 3 summarizes the RR for LBP between the different occupational groups (AU, CD, TD and total drivers) according to initial LBP severity (categories 1–3). The RR for LBP was higher but non-significant amongst CD when compared to AU in categories 2 and 3. The RR for LBP was lower amongst total drivers and TD compared to AU in category 1 (RR = 0.62 and RR = 0.49, respectively, *p* < 0.001). No significance was observed when comparing heavy-vehicle (TD) to light-duty drivers (CD).

**Table 3:** Relative risk (RR) and *p*-values for low back pain (LBP) among different occupational groups by severity of disease at recruitment

Group comparison	Category 1	Category 2	Category 3
Car driver vs. administrative	1.00 (NS)	1.13 (NS)	1.35 (NS)
Truck driver vs. administrative	**0.49 (0.40–0.60,** ***p* < 0.001**)	1.03 (NS)	0.82 (NS)
Total drivers vs. administrative	**0.62 (0.527–0.726,** ***p*** **< 0.001)**	1.07 (NS)	1.07 (NS)
Truck driver vs. car driver	0.49 (NS)	0.92 (NS)	0.61 (NS)

Category 1 – No history of LBP

Category 2 – Mild LBP

Category 3 – LBP with mild clinical/radiographic findings

Bold data already signifies a *p* < 0.05 i.e. significant finding

## Discussion

In this research we investigated the incidence and recrudescence of LBP amongst administrative vs. driving professions in a population of young male soldiers from their time of recruitment to service for a follow up period of three years. The study concluded 80,599 soldiers with a cumulative 241,797 person-years of data.

We found that in category 1, the incidence of LBP in AU was twice higher than that of TD (*p* < 0.0001). There was no significant difference in incidence rates by profession amongst the other categories (2–3). The risk for LBP exacerbation was associated with the initial state of back pain in soldiers from all categories. The exacerbation rate for soldiers with a history of LBP was 1.38–3.83 times higher in comparison to soldiers without a history of LBP.

The total LBP incidence rate was 0.65%. The yearly incidence of LBP in the US population is 1–5% [21]. Knox et al. [18] investigated 13,754,261 person-years of data (557,059 subjects) in the US Army. The overall unadjusted incidence rate of LBP was 40.5 per 1000 person-years (4%). The incidence rate of LBP in people younger than 20 was 40.2 per 1000 person-years (4%); a higher incidence was found among recruits (4.8%). Mattila et al. [22] demonstrated that the hospitalization incidence due to unspecified LBP among recruits was 19.1 per 1000 person-years (2%), and 7.8 per 1000 person-years (0.8%) due to lumbar disc disorders. Waterman et al. [21] estimated an incidence rate of acute LBP in the US of 1.39 per 1000 person-years (0.14%). Bar Dayan et al. [17] found an incidence of LBP of 0.45% in a similar recruits-group study in the IDF. In Ernat et al. [23] the unadjusted rate of acute LBP for enlisted infantrymen was 35.2 per 1000 person-years (3.52%). In our study the overall incidence rate was 0.65%. These differences are explained by this study’s endpoint (Category 4) which represents severe LBP after a full assessment of symptoms including clinical and imaging findings rather than other studies’ reporting of initial events of LBP [18, 23] or first hospitalizations [22]. Studies with a similar design to ours exhibited similar results [17, 18].

The RR for the development of severe LBP with clinical and imaging findings (category 4) in soldiers who suffer from LBP occasionally without clinical findings (category 2) and in soldiers who suffer from LBP with mild clinical or imaging findings (category 3) was RR = 1.38 (95% CI 1.17–1.64) and RR = 3.83 (95% CI 3.12–4.71) respectively (Table 2). When we examined the recurrence risk in the same categories in different professions we found the same trend in most of the categories. For example, the recurrence risk in the AU in similar categories (the RR of exacerbation to category 4 from category 2 and from category 3) was RR = 1.28 (95% CI 1.07–1.53) and RR = 3.55 (95% CI 2.85–4.42) respectively (Table 2). These findings raise the question of whether a history of LBP is a risk factor for LBP in new workers. In their systematic review, da Silva et al. describe the lack of accepted estimates for the risk of recurrence of LBP largely due to poor-quality study design and bias; they report an estimate proportion for a 1-year recurrence as 33% [24]. Himmelstein et al. [25] reported on risk evaluation for LBP at preplacement screening in the late 80s. The most sensitive indicator was past history of low back pain; Other indicators included number of sick days, frequency of symptoms etc. Videman [26] investigated the prevalence of LBP from entering the nursing school and found that LBP at entry was a predictor for back-related pain and disability (OR = 7.1). Ryan et al. [27] found that history of LBP was a predictor for back-related absence and disability.

To sum, our results support the concept that a history of LBP is a risk factor for LBP.

In this research we found that the incidence of LBP in driving professions was lower than in AU (RR = 0.49 vs. RR = 0.62, *p* < 0.001). These results contradict other studies which showed that professional drivers comprise a high-risk group for LBP [4, 8–17, 19]. Most of these studies are based on questionnaires [9–13, 16] and only a few are based on medical records [14] or database [18, 19]. In the database studies, spinal disorders were more common among professional drivers with a standardized hospitalization ratio of 119 [14] with a higher incidence rate ratio for LBP of 1.15 [19], representing lower ratios than in questionnaires studies. The basis for our research is the medical records, which document chronic and continuous LBP with significant imaging and neurological findings. Our rates were therefore lower (0.4–0.8%) than similar studies based on questionnaires (5–7%) and similar to database studies [14, 17, 19]. Other differences in our study are the drivers’ age and experience. In other studies, the average reported age is 40–50-year-olds with a driving experience of 10 years; Our study includes 18–21-year-olds with little driving experience. We believe that the younger age of our drivers with their respective driving experience affect the risk of exacerbating back problems. We therefore think that driving at a young age does not necessarily modify the prognosis of LBP in the first years of professional driving. We presume that among professional drivers, in order for driving to have an adverse effect on back disorders, a prolonged exposure period may be required.

The weaknesses of our research are the short follow-up period (3 years) and the inability to control for potential LBP confounders such as motivation and other psychological factors. The strengths of our study include the large population (over 80,000) and the precise and standardized criteria for LBP [20]. To overcome the potential age confounder for LBP, we chose a homogenous group of subjects within the ages of 18–21.

## Conclusions

To conclude, this study included 80,000 soldiers who represent a population of driving and administrative occupations aged 18–21. The significant risk factors for developing LBP were a positive history of previous LBP and presence of LBP symptoms at the start of work. A correlation was identified between severity of LBP at the initial examination and further exacerbation of LBP in all examined occupations. A higher incidence of LBP was found among administrative occupations compared to driving occupations with the ensuing conclusions that driving as a profession, at this age group and during this period of exposure (3 years), are not risk factors for LBP. This finding may be explained by factors unaccounted for in our study thus requiring further studies on the matter.

## Abbreviations

AU: Administrative Units
CD: car drivers
CU: Combat Units
IDF: Israel Defense Forces
LBP: Low Back Pain
MU: Maintenance Units
RR: Relative Risk
TD: Truck drivers
